# Keeping up with sea-level rise: Carbonate production rates in Palau and Yap, western Pacific Ocean

**DOI:** 10.1371/journal.pone.0197077

**Published:** 2018-05-08

**Authors:** Robert van Woesik, Christopher William Cacciapaglia

**Affiliations:** Department of Biological Sciences, Florida Institute of Technology, West University Blvd., Melbourne, Florida, United States of America; Universita degli Studi di Genova, ITALY

## Abstract

Coral reefs protect islands from tropical storm waves and provide goods and services for millions of islanders worldwide. Yet it is unknown how coral reefs in general, and carbonate production in particular, will respond to sea-level rise and thermal stress associated with climate change. This study compared the reef-building capacity of different shallow-water habitats at twenty-four sites on each of two islands, Palau and Yap, in the western Pacific Ocean. We were particularly interested in estimating the inverse problem of calculating the value of live coral cover at which net carbonate production becomes negative, and whether that value varied across habitats. Net carbonate production varied among habitats, averaging 10.2 kg CaCO_3_ m^-2^ y^-1^ for outer reefs, 12.7 kg CaCO_3_ m^-2^ y^-1^ for patch reefs, and 7.2 kg CaCO_3_ m^-2^ y^-1^ for inner reefs. The value of live coral cover at which net carbonate production became negative varied across habitats, with highest values on inner reefs. These results suggest that some inner reefs tend to produce less carbonate, and therefore need higher coral cover to produce enough carbonate to keep up with sea-level rise than outer and patch reefs. These results also suggest that inner reefs are more vulnerable to sea-level rise than other habitats, which stresses the need for effective land-use practices as the climate continues to change. Averaging across all reef habitats, the rate of carbonate production was 9.7 kg CaCO_3_ m^-2^ y^-1^, or approximately 7.9 mm y^-1^ of potential vertical accretion. Such rates of vertical accretion are higher than projected averages of sea-level rise for the representative concentration pathway (RCP) climate-change scenarios 2.6, 4.5, and 6, but lower than for the RCP scenario 8.5.

## Introduction

The recent increase in the frequency and intensity of thermal-stress events has resulted in coral bleaching and coral mortality, which has subsequently changed the composition of many reef assemblages worldwide [1–5]. Changes in reef composition and loss of major reef-building corals reduces the potential of coral reefs to accrete calcium carbonate, and impairs their capacity to keep up with sea-level rise [6]. Given the suite of modern circumstances that are detrimental to coral reefs, one of the central questions in contemporary marine ecology is: Where will coral reefs be able to accumulate carbonate fast enough to ‘keep up’ [7] with projected sea-level rise, as the ocean temperatures continue to increase and as storm patterns change from their historical trajectories [8]?

For the last 5000 years, reef flats in the central and western Pacific Ocean have been constrained at modern sea level by aerial exposure during low-water-spring tides. Because of the relatively stable sea level for over five millennia, reef flats have existed largely in a dormant state [9,10] and the expansion of reefs only occurred by gradual carbonate accumulation along the reef edges. Such lateral progradation occurred at locations where the rates of local production of calcium carbonate exceeded rates of local destruction [11–18]. Recently, using high-precision U-series aging of geological cores, Roff *et al*. [10] estimated that over the last 1000 years, reef slopes along the inner Great Barrier Reef have grown rapidly, between 3.5 and 35 mm per year, with average growth rates of 11.5 ± 1.1 mm per year. Montaggioni [11] showed evidence, also from geological cores, that lateral accretion of reef slopes was historically faster than vertical accretion. Modal vertical accretion rates of reefs with framework-dominated corals occurred at 6–7 mm year through the Holocene [11]. Yet arborescent-acroporid rich assemblages on Indo-Pacific reefs accreted vertically at rates of up to 20 mm per year. Although averaging over geological time-periods may conceal the capacity of modern reefs to keep up with modern sea-level rise [19], these rates agree remarkably with recent measurements of vertical extension of *Porites* microatolls (~ averaging 11.8 ± 2.7 mm y^-1^) in Palau [20]. Still, reef growth is more complex than simply a consequence of coral growth. Reefs grow by the incremental buildup of calcium carbonate from calcifying corals, coralline algae, and from sediment, and erode by physical (e.g., cyclones), chemical (e.g., ocean acidification), and biological (e.g., fishes, echinoids, and boring infauna) processes [11,16, 21–24]. Gradual accumulation of calcium carbonate develops reef structures over geological time, benefiting coastal residents worldwide by protecting tropical island nations from storm waves [25]. Losing coral reefs as wave barriers is a critical threat to island nations that lie close to modern sea level [25, 26], especially as the sea level continues to rise.

Historically, the maximum rate of carbonate production in the Pacific Ocean was estimated at 10 kg CaCO_3_ m^-2^ y^-1^, which was previously translated to approximately 7 mm of reef growth per year [11,13,27]. A ‘healthy’ coral reef was thought to accumulate, on average, ~4 kg CaCO_3_ m^-2^ y^-1^, which was translated to approximately 3 mm of reef growth per year, and a reef with low coral cover, <10%, has been estimated to accrete less than 1 kg CaCO_3_ m^-2^ y^-1^, which was translated to approximately 1 mm of reef growth per year [11,13,27]. However, past estimates of rates of carbonate production mainly used the *in situ* alkalinity-anomaly technique [27], which measured the change in total alkalinity across a reef, over several hours. Extrapolating chemical flux, acquired in less than a few hours, to predict rates of annual reef growth is problematic because those measurements do not consider diel, weekly, or even seasonal fluctuations.

Recently, Perry *et al*. [6] calculated *in situ* rates of carbonate production, as a product of the cumulative sum of linear extension and density of reef accretors minus estimates of bioerosion. These estimates provide a useful approximation of spatial variation in carbonate production rates [6,28]. The modern rates of accretion estimated by Perry *et al*. [17] showed that most reefs in the Caribbean have low rates of modern carbonate production, averaging 3.5 kg CaCO_3_ m^-2^ y^-1^, with some reefs showing negative carbonate budgets (i.e., many reefs were undergoing net erosion). Similarly, studies on carbonate budgets of 28 sites across the Chagos Archipelago, in the Indian Ocean, showed production rates at 3.7 kg CaCO_3_ m^−2^ yr^−1^, with higher rates of 8.4 kg CaCO_3_ m^−2^ yr^−1^ for *Acropora*-dominated reefs [29]. Perry and Morgan [29] also showed the sensitivity of carbonate budgets to thermal stress, reporting a shift in reef accretion to -3 kg CaCO_3_ m^−2^ yr^−1^ on Maldivian reefs immediately after a coral bleaching event.

Here we take a field-based approach to quantify the different accretors and eroders in Palau and Yap to derive spatial estimates of net-carbonate production rates across different habitats. Based on geological evidence, we hypothesize that there will be spatial differences in reef-building capacity across habitats on reefs in both Palau and Yap, with windward, eastern reefs producing less carbonate than leeward, western reefs, and inner reefs producing the lowest amount of carbonate. We were particularly interested in estimating the value of live coral cover at which net carbonate production becomes negative across the different habitats. We were also interested in examining the impact on carbonate budgets four years after two sequential cyclones passed near Palau in 2012 and 2013. Tropical cyclones have recently become more common in the tropics [30,31], where they were historically considered rare events [8]. Specifically, the objectives of this study were to: (1) quantify potential net carbonate production rates across different reef habitats in Palau and Yap to determine which habitats are most likely to keep up with sea-level rise, and (2) determine the value of live coral cover at which net carbonate production became negative in each habitat.

## Methods

### Field surveys

**Fig 1:**
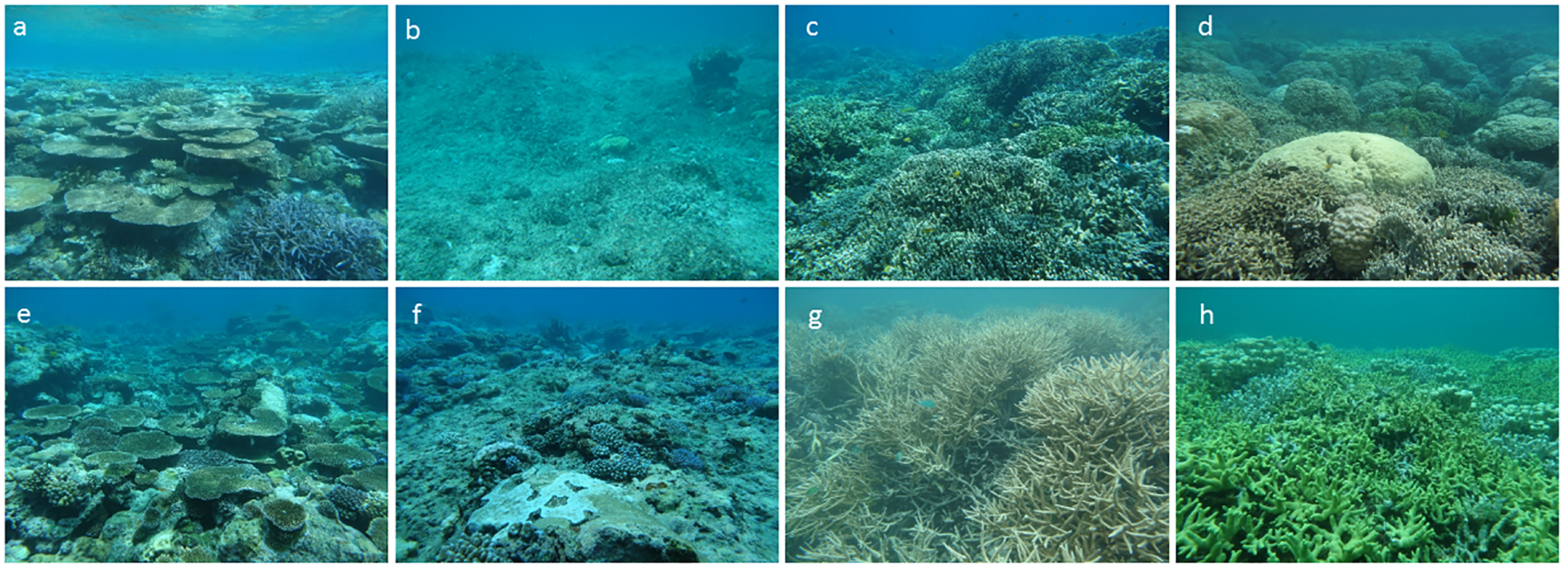
Representative images of reef habitats in Palau and Yap. Palau reefs, top row: a) outer western reef, b) outer eastern reef, c) patch reef, d) inner reef. Yap reefs, bottom row: e) outer western reef, f) outer eastern reef, g) a rare patch reef, and h) inner reef.

We used a stratified random sampling approach to survey the reefs of Palau (7°30' N, 134°30' E) and Yap (9°32' N, 138°7' E) (Figure A in S1 File), by randomly selecting 24 study sites at each island using the package ‘sp’ [32] in R [33]. We stratified the sites in Palau by outer reefs (8), patch reefs in the lagoon (10), and inner reefs (6) (Fig 1). In Yap, which does not have an extensive lagoon and only supports a few rare patch reefs, we stratified the sites by outer reefs (10) and inner reefs (14). The allocation of sites per strata were dependent on the reef area. We were particularly interested in determining the potential of shallow-water reef carbonate production, and therefore focused our surveys between 2–5 m.

At each site we quantified reef composition. Corals were identified to species level, except encrusting *Montipora* and massive *Porites*, which were identified to life-forms. Crustose coralline algae, macroalgae, sponges, ascidians, tunicates, and other benthic components were identified to the highest taxonomic resolution that was possible in the field. At each site we laid six, 10 m long transect tapes, which followed the contours of the reef substrate. The tapes were placed approximately 2 m between the end of one tape and the start of the next tape. Using these transects we applied the line-intercept method [34] to quantify the planar chord length of each benthic component to the nearest centimeter. At each site we also ran six, 10 m transect lines horizontally along the substrate, above the transects that followed the reef contours. These horizontal lines were used primarily to approximate rugosity, by measuring the length difference between the horizontal and the contour-following lines. Echinoids were counted 30 cm along either side of each 10 m horizontal transect, identified as *Diadema*, *Echinometra*, or ‘*Other*’ urchins, and each echinoid test diameter was measured to the nearest millimeter. Fishes were videoed along six, 30 m long by 4 m wide transects. The herbivorous parrotfishes in the videos were subsequently analyzed for size (cm) and identity.

### Calculating net carbonate production

One of the major tasks of this work was to determine the contribution of the various components on each reef to potential carbonate production. Live coral cover was calculated as the sum of live coral cover for each transect. Net carbonate production (kg CaCO_3_ m^-2^ yr^-1^) was considered as:

$${Reef\;accretion}_{i}={Cal}_{i}+{sgn(x)Sed}_{i}–{Eros}_{i},$$ (1)

where *Cal* is the rate of calcification by reef-building corals and coralline algae, at a site *i*, sgn is positive when local sedimentation (*Sed*) is low, and negative when local sedimentation is high, and *Eros* is the rate of erosion (after [35]). Gross carbonate production was estimated in units of kg CaCO_3_ m^-2^ yr^-1^, and was summed across all calcifying species of reef accretors, where *Cal* was estimated as:

$${Cal}_{i}=r_{i\;}\;*\;\{\Sigma \;[(m_{i,j}\;*\;x_{i,j}\;/\;100)\;*\;d_{i,j}\;*\;g_{i,j}\;*\;10]+{ca}_{i}\},$$ (2)

where *r* is the averaged rugosity of site *i*, *m* is the morphological adjustment coefficient for coral morphologies (Table A in S1 File) at site *i* for species *j*, *x* is the mean percent planar cover of carbonate-accreting species *j* at site *i*, *d* is the density (g cm^-3^) of species *j* at site *i* (Table B in S1 File), and *g* is the vertical growth rate (cm year^-1^) of species *j* at site *i*. Ten was inserted in the model as an adjustment coefficient to set the units at kg CaCO_3_ m^2^ yr^-1^, and *ca* is the contribution of coralline algae at site *i* to reef accretion, which was defined as:

$${ca}_{i}=0.018\;*\;(p{ca}_{i})\;*\;10,$$ (3)

where *pca* is the planar cover of coralline algae at site *i*, 0.018 is the average gross carbonate production of coralline algae (g cm^-2^) [6], and 10 is the conversion between g cm^-2^ and kg m^-2^.

Reef erosion was broken down into three major components, defined as:

$$Eros_{i}=\Sigma (parrotfish_{i,j}+urchin_{i\;,j})+\;macroboring,$$ (4)

where *parrotfish* is the biological erosion caused by parrotfish at site *i* by species *j*, *urchin* is erosion caused by sea urchins at site *i* by species *j*, and *macroboring* is the erosion caused by macroboring organisms. The erosion caused by parrotfish was defined as:

$${parrotfish}_{i}=\Sigma ({vol}_{i,j,n}\;*\;{sp}_{i,j,n}\;*\;{br}_{i,j,n})\;*\;D_{i}\;*\;365\;*\;0.001,$$ (5)

where *vol* is the bite volume (cm^3^) for individual *n* of species *j* at site *i*, *sp* is the proportion of bites that leave a scar at site *i* for individual *n* of species *j*, *br* is the bite rate (bites day^-1^) at site *i* of species *j* for individual *n*, *D* is the average density of corals at site *i*, 365 is used to convert erosion rate to years, and 0.001 is to convert g to kg. In Eq 5, *vol* was defined as:

$${vol}_{i,j,n}=\frac{e^{1.32+0.06\;*\;{\;length}_{i,j,n}}}{1000},$$ (6)

where, *length* is the length (cm) of parrotfish *n* of species *j* in site *i*, the constants 1.32 and 0.06 were generated from a regression of data from Ong & Holland [36], and 1000 was used to convert from mm^3^ to cm^3^. In Eq 5, *sp* is the scar proportion of fish *n* of species *j* at site *i*, defined as:

$${sp}_{i,j,n}=1/[1+e^{-(-\;2.46+0.089\;*\;{length}_{i,j,n})}],$$ (7)

following a regression from data gathered from Bonaldo & Bellwood [37] and [36], where *length* is the length (cm) of fish *n* of species *j* at site *i*. In Eq 5, *br* is the bite rate (bites day^-1^) at site *i* of species *j* for individual *n*, defined as:

$${br}_{i,j,n\;}=60\;\{\left[\left(4.31+{brc}_{i,j}-\;0.36)-(0.045\;*\;reeftime\;*\;{length}_{i,j,n}\right)\right]\},$$ (8)

where *brc* is the bite rate constant derived from data provided by Peter Mumby (pers. comm.) for species *j* at site *i*, *reeftime* is the length of time fishes spend grazing on the reef estimated at 9 hours a day, *length* is the length (cm) of fish *n* of species *j* at site *i*, 60 is to convert the units from minutes to hours, and all other constants were derived from bite rate data. The bioerosion (kg CaCO_3_ m^-2^) caused by echinoids was defined as:

$${urchin}_{i}=\Sigma ({Diadema}_{i,n}+{Echinometra}_{i,n}+{Other\;urchins}_{i,n}\;),$$ (9)

where *Diadema* is the erosion caused by species in the genus *Diadema* at site *i* for individual *n*, *Echinometra* is the erosion caused by species within the genus *Echinometra* at site *i* for individual *n*, *Other urchins* is the erosion caused by echinoid species not in the genera *Echinometra* or *Diadema*. *Diadema* was defined by a function from Januchowski-Hartley et al. [38] as:

$${Diadema}_{i,n}=(0.000001\;*\;{{diameter}_{i,n}\;}^{3.42})\;*\;0.365\;*\;0.57,$$ (10)

where *diameter* is the diameter (cm) of the *Diadema* test. The function for *Echinometra* follows an equation from Januchowski-Hartley et al. [38] and was defined as:

$${Echinometra}_{i,n}=(0.0004\;*\;{{diameter}_{i,n}\;}^{1.98})\;*\;0.365\;*\;0.57,$$ (11)

where *diameter* is the diameter (cm) of the *Echinometra* test. *Other urchins* also follows an equation from Januchowski-Hartley et al. [38] and was defined as:

$${Other\;urchins}_{i,n}=(0.0001\;*\;{{diameter}_{i,n}\;}^{2.32})\;*\;0.365\;*\;0.57,$$ (12)

where *diameter* is the diameter (cm) of the echinoid test. We were particularly interested in the capacity of clinoid sponges to bioerode carbonate substrate, whereas other macroborers such as polychaetes, crustaceans, sipunculids, and molluscs [14] were more inconspicuous during our surveys. Therefore, *macroboring* was defined as:

$${macroboring}_{i}=plamc_{i}\;*\;mec,$$ (13)

where *plamc* is the mean planar cover of macroboring organisms for site *i*, and *mec* is a macroboring erosion constant, for which we use a conservative estimate of 10 kg CaCO_3_ m^-2^ y^-1^ for clinoid sponges (after [14]).

Carbonate sediment can contribute to reef accretion [39], as outlined in Eq (1). However, we noticed no obvious direct terrestrial sedimentation at the surveyed sites in both Palau and Yap, therefore we consider that *Sed* in Eq (1) as positive, irrespective of whether the sediment was bioerosion-derived or direct. We estimated that the positive contribution of sediment to carbonate production was no more than 0.4 kg CaCO_3_ m^-2^ y^-1^ [11, 39].

We estimated reef accretion by solving Eqs (1) to (13) for each transect, and plotted the estimated rates of reef accretion across the spatial fields. We firstly used semivariograms to estimate the extent of spatial autocorrelation, and examined the spatial data for isotrophy (i.e., directionality). We then used the information from the semivariograms and the isotrophy to run a series of ordinary kriging analyses to interpolate the data across the spatial fields of both islands. To convert rates of reef accretion to vertical reef growth we used:

Verticalreefgrowth=Cp+Cp(Cp*alpha), (14)

where *Cp* is carbonate production and *alpha* is an estimated coefficient (Figure B in S1 File).

### Data analysis

We were particularly interested in estimating the inverse problem [35] of calculating the value of live coral cover at which net carbonate production becomes negative for the different habitats. To derive these estimates, and provide a measure of uncertainty around the values (i.e., 95% credible intervals), we used an additive mixed effects model in a Bayesian framework [40] using the following:

$$G_{i,j,k}=Beta+f({Live\;cover\;cover}_{i,j})+\;{Habitat}_{i,j}+a_{i}+{error}_{i,j},$$ (15)

where *G*_*ijk*_ is the *k*th observation (transect) of net carbonate production at site *j* in country *i*, *f*(*Live coral cover*) is a smoothing function. We used an O’Sullivan spline [41] for the smoother with five knots [40]. *Habitat* is the covariate of interest, *a*_*i*_ is a random intercept for each country (Palau and Yap), for which we used a normal distribution, and *error*_*ij*_ is the error term for the residuals, for which we also used a normal distribution. We used multivariate normal diffuse and normal diffuse priors throughout the analysis, assuming no prior information was known [40]. The models were coded in JAGS [42], which were run through R [33]. (Note that the Palau data were collected under the auspices of the Palau International Coral Reef Center research permit, and the Yap data were collected under auspices of collaboration with YapCAP. We did not sample or involve any endangered or protected species. All the data and the R code for Eqs 1 to 14 are available in the supplementary online document; the data are also deposited at: https://www.bco-dmo.org/award/709533).

## Results and discussion

**Fig 2:**
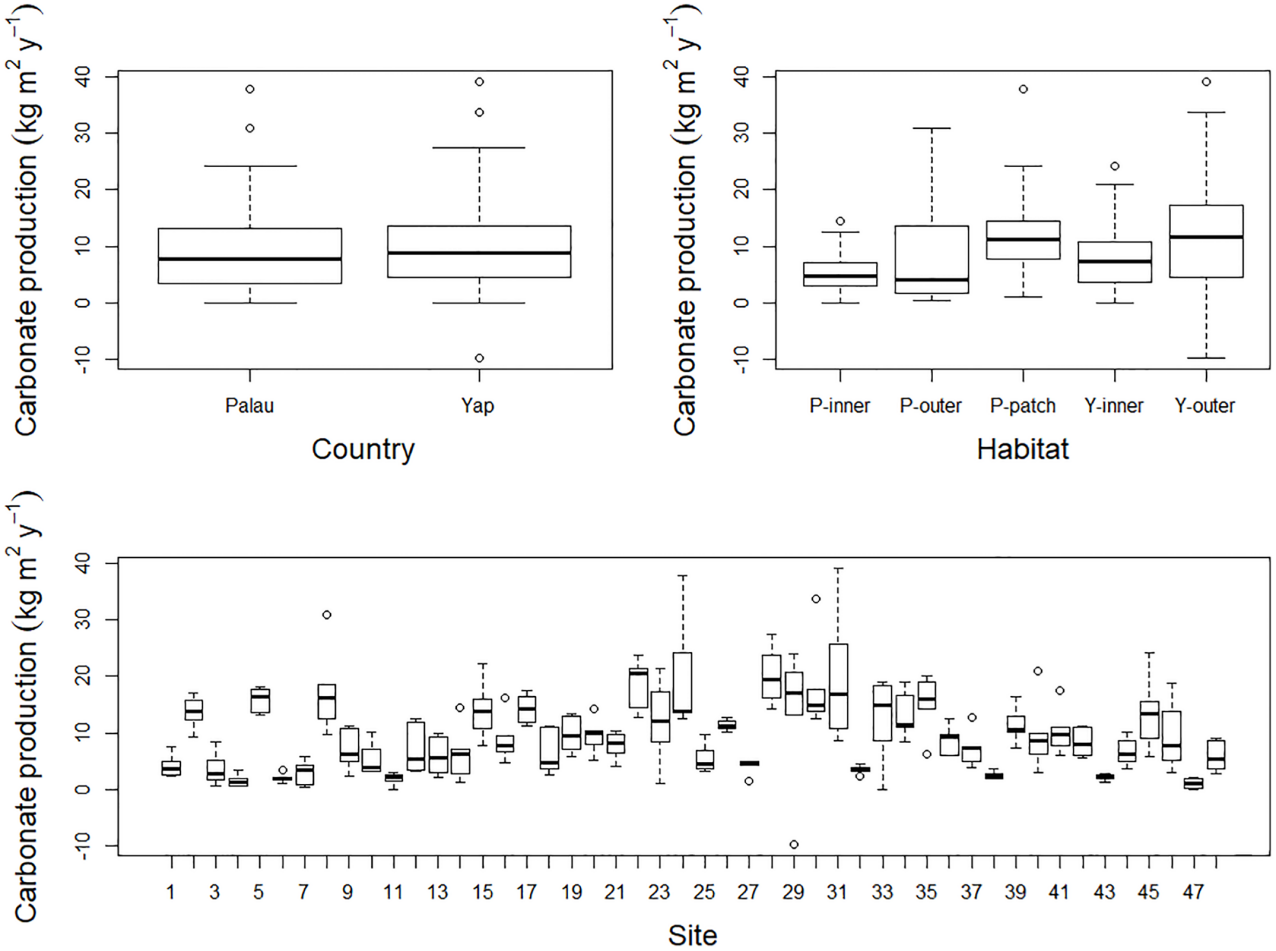
Summary of carbonate production across all sites. Summary of rates of carbonate production (kg CaCO_3_ m^-2^ yr^-1^) conditional on country, habitat, and site, where the thick horizontal lines are the medians, the box surrounding the medians are the first and third quartiles, the whiskers identify the range of the data, and the circles identify outliers. P-inner refers to Palau inner reefs, P-outer refers to Palau outer reefs, P-patch refers to Palau patch reefs, Y-inner refers to Yap inner reefs, and Y-outer refers to Yap outer reefs.

**Fig 3:**
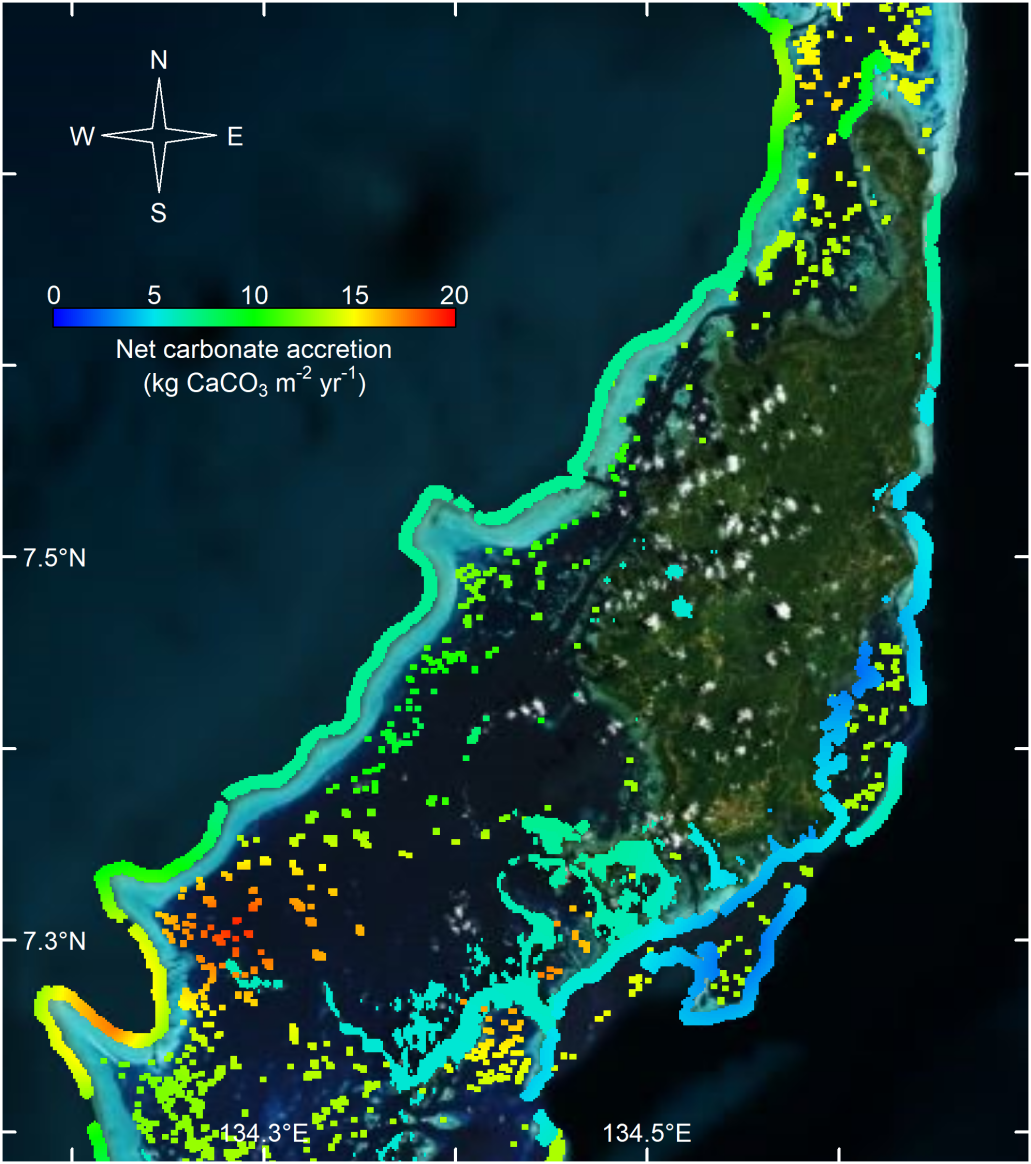
Net carbonate production for Palau. Net carbonate production rates (kg CaCO_3_ m^-2^ yr^-1^), kriged for Palau. Red indicates high rates of net carbonate production and blue indicates low rates of net carbonate production.

Although the rates of net carbonate production were similar on Palau and Yap, the rates were considerably different among habitats and across sites. In Palau, the estimated rates of net carbonate production were highest on the western outer reefs, averaging 13.1 kg CaCO_3_ m^-2^ yr^-1^, and on the western and northern patch reefs, averaging 12.7 kg CaCO_3_ m^-2^ yr^-1^ (Table 1, Figs 2 and 3). The eastern outer reefs of Palau, still recovering from cyclones in 2012 and 2013, had the lowest rates of carbonate production, averaging 2.8 kg CaCO_3_ m^-2^ y^-1^ (Figs 2 and 3). The estimated rates of carbonate production on Palau’s inner reefs averaged 5.8 kg CaCO_3_ m^-2^ yr^-1^, although some sites had rates that were considerably lower (Figs 2 and 3).

**Table 1:** Summary of calcification and erosion rates for the habitats in Palau and Yap (kg CaCO_3_ m^-2^ yr^-1^), where CI are the 95% Confidence intervals, and n/a is not applicable.

	***Palau***				
	**Outer (CI) n = 8**			**Inner (CI) n = 6**	**Patch (CI) n = 10**
	**All Outer** **n = 8**	**Western Outer n = 4**	**Eastern** **Outer n = 4**		
*Gross calcification*	7.60 (4.66)	12.72 (5.79)	2.48 (0.90)	5.46 (1.48)	12.32 (2.83)
*Erosion*	0.08 (0.01)	0.025 (0.02)	0.02 (0.02)	0.06 (0.01)	0.06 (0.00)
*Net calcification*	7.98 (4.67)	13.09 (5.80)	2.86 (0.90)	5.80 (1.48)	12.66 (2.83)
	***Yap***				
	**Outer (CI) n = 10**			**Inner (CI) n = 14**	**Patch (CI)**
		**Western Outer n = 6**	**Eastern** **Outer n = 4**		
*Gross calcification*	12.53 (4.11)	15.08 (6.09)	11.44 (5.65)	8.26 (2.19)	n/a
*Erosion*	0.47 (0.78)	1.04 (1.96)	0.08 (0.02)	0.09 (0.03)	n/a
*Net calcification*	12.47 (3.89)	14.07 (5.32)	11.40 (5.65)	8.57 (2.19)	n/a
	***Mean values for Palau and Yap***				
*Gross calcification*	10.34 (3.21)			7.40 (1.68)	
*Erosion*	0.27 (0.43)			0.06 (0.01)	
*Net accretion*	10.47 (3.09)			7.73 (1.68)	

**Fig 4:**
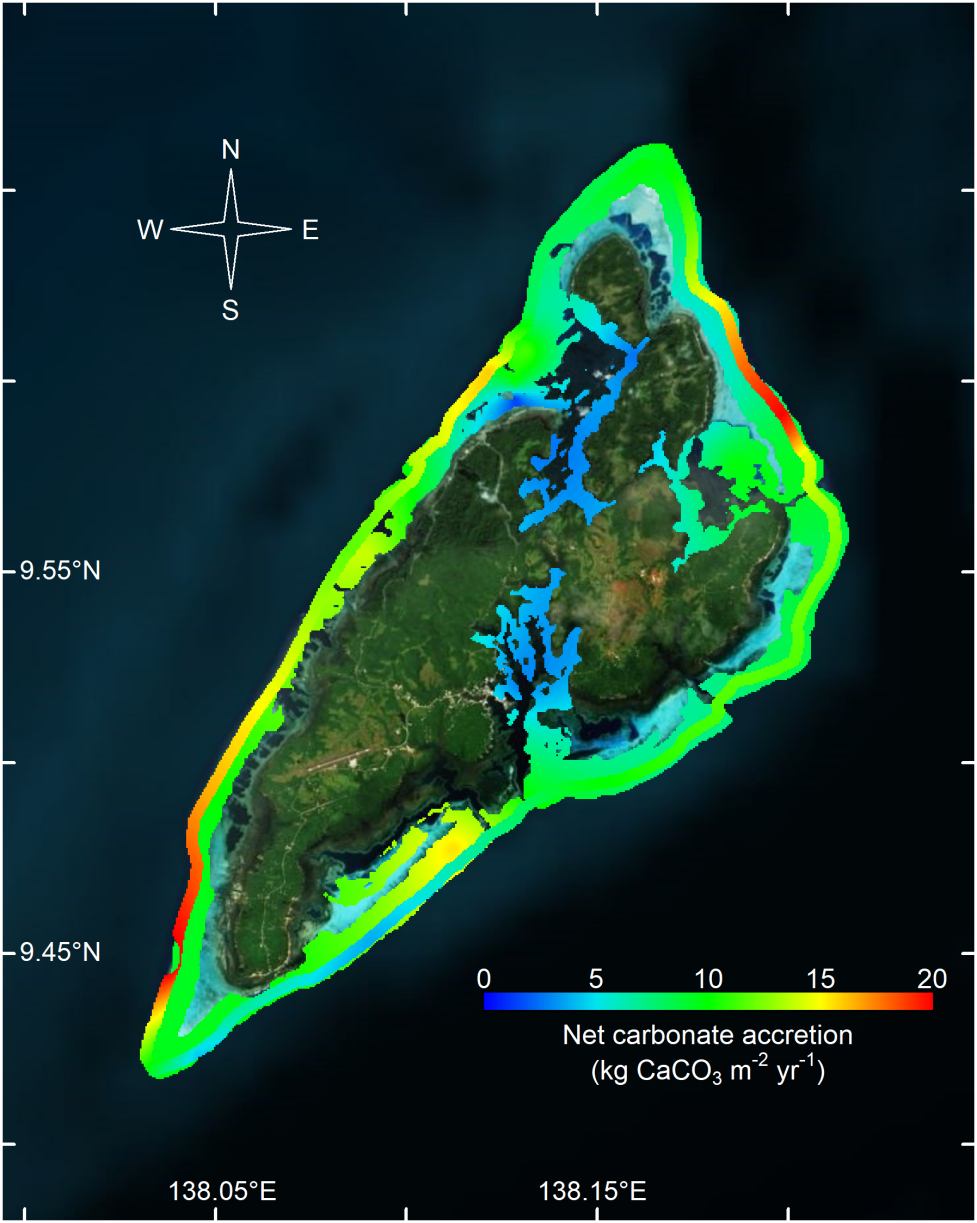
Net carbonate production for Yap. Net carbonate production rates (kg CaCO_3_ m^-2^ yr^-1^), kriged for Yap. Red indicates high rates of net carbonate production and blue indicates low rates of net carbonate production.

In Yap, the estimated rates of net carbonate production were similar to rates in Palau, with highest rates recorded on the western outer reefs, averaging 14.1 kg CaCO_3_ m^-2^ yr^-1^. The eastern outer reefs of Yap showed moderate rates of net carbonate production, at 11.4 kg CaCO_3_ m^-2^ yr^-1^, except along the southeastern slope, where rates were as low as 5 kg CaCO_3_ m^-2^ yr^-1^ (Figs 2 and 4). The inner reefs of Yap had net carbonate production rates at 8.6 kg CaCO_3_ m^-2^ yr^-1^ (Table 1), except within the nearshore inlets, where rates were < 2 kg CaCO_3_ m^-2^ yr^-1^ (Fig 4).

On both islands, in the surveyed transects, there were around 100 coral species contributing to carbonate production, although 10% of the coral species contributed more than 75% of overall net carbonate accretion in Palau and more than 65% in Yap (Figure C in S1 File). Although similar rates of carbonate production were measured on both islands, the main reef-building corals differed between Palau and Yap. In Palau, the main reef-building corals were *Porites rus*, *Porites cylindrica*, *Porites lobata*, and *Acropora formosa* (*muricata*), whereas in Yap, the main reef building corals were *Porites cylindrica*, *Acropora formosa* (*muricata*), *Acropora palifera*, and *Porites lobata* (Figure C in S1 File).

Overall, the highest rates of erosion were a consequence of grazing by herbivorous fishes. Unexpectedly, the highest rates of erosion by fishes were at sites where gross carbonate production rates were also highest. The majority of carbonate removal by herbivorous fishes in Palau was measured on the western outer reefs, at localities where carbonate production rates were also high (Figures D and E in S1 File). *Chlorurus sordidus* and *Scarus dimidiatus* were responsible for most of the inner reef erosion (Figure D in S1 File), although both species were ubiquitous across both islands (Figure E in S1 File). The maximum rate of carbonate removal by herbivorous fishes in Yap was much higher than in Palau (Figures D and F in S1 File). The higher rates were localized, however, and were mainly caused by large *Bolbometopon muricatum*, particularly in the northwest (Figure F in S1 File). Unlike in Palau, herbivorous fish erosion in Yap influenced rates of net carbonate production, particularly in those areas supporting dense schools of large *B*. *muricatum*. Even ignoring the effect of the *B*. *muricatum*, the rates of erosion by herbivorous fishes tended to be high in northwestern Yap.

The removal of carbonate by echinoids was highest on the outer reefs in both Palau and Yap (Figure G in S1 File), and coincided with areas of lowest rates of carbonate production. In Palau, echinoid erosion was highest on the eastern outer reefs, where there was low coral cover relative to the other outer reefs. In Yap, the highest rates of erosion were in the southeast (Figure G in S1 File). Overall, the rates of echinoid erosion in Yap were an order of magnitude greater than the rates in Palau (Table C in S1 File), and were similar to rates of erosion caused by herbivorous fishes in Palau. Bioerosion caused by macroboring organisms was minimal on both islands, and estimated at 0.058 and 0.044 kg CaCO_3_ m^-2^ yr^-1^ in Palau and Yap, respectively (Table 2).

**Table 2:** Species contributions to macro-bioerosion in Palau and Yap (kg CaCO_3_ m^-2^ yr^-1^).

Macroborer	Palau	Yap
Encrusting sponge	0.044	0.041
*Cliona* spp. sponge	0.002	0.003
*Turpios* spp. sponge	0.013	0
Total	0.058	0.044

**Fig 5:**
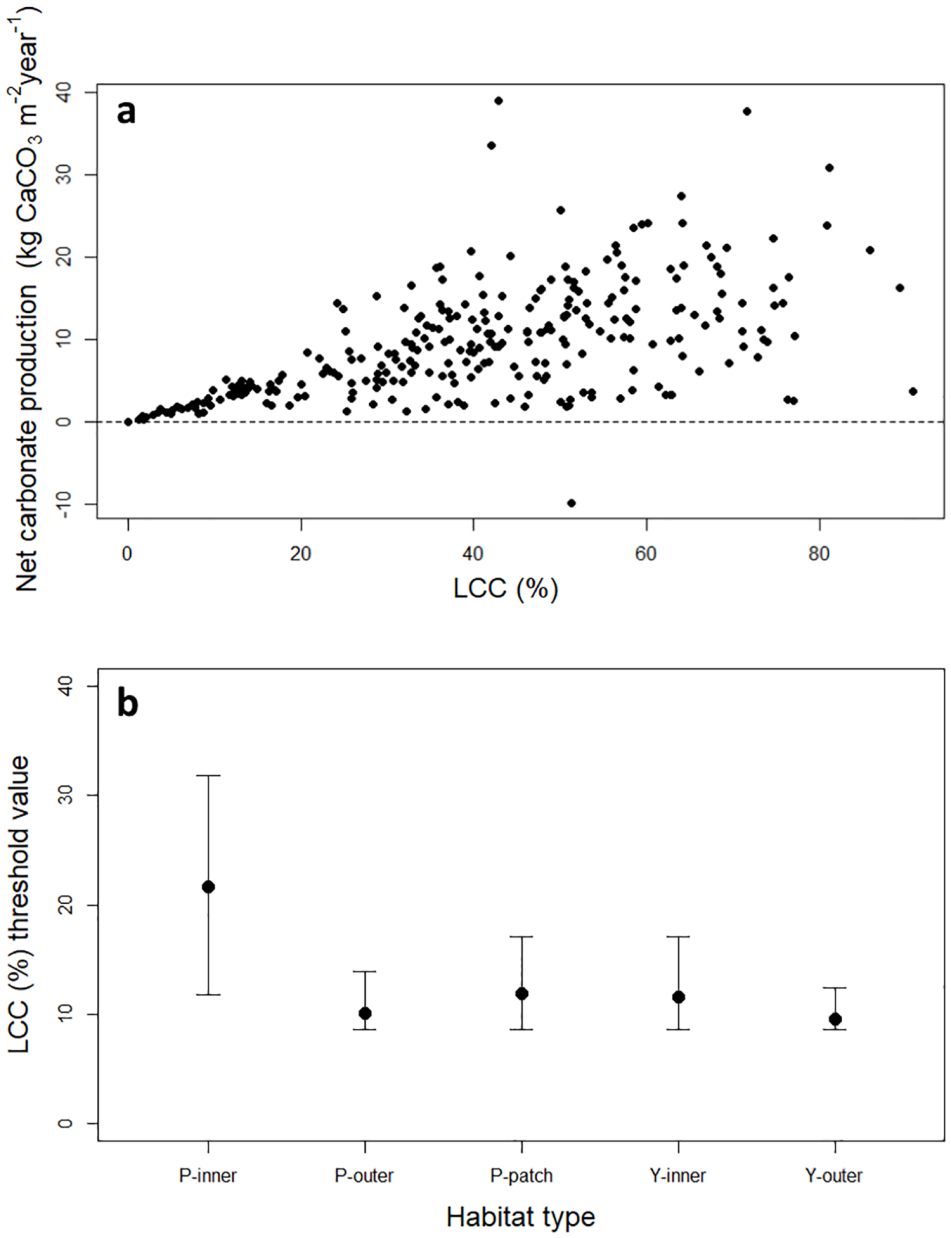
Live coral cover (LCC) threshold values. **a.** Net carbonate production (kg CaCO_3_ m^-2^ y^-1^) against live coral cover (%) at all sites in Palau and Yap. **b.** Threshold values of live coral cover at which net carbonate production became negative for the different reef habitats in Palau and Yap.P-inner refers to Palau inner reefs, P-outer refers to Palau outer reefs, P-patch refers to Palau patch reefs, Y-inner refers to Yap inner reefs, and Y-outer refers to Yap outer reefs.

Our study found that shallow-water coral reefs of Palau and Yap in the western Pacific Ocean had high rates of carbonate production, averaging 9.7 kg CaCO_3_ m^-2^ y^-1^. The value of live coral cover at which net carbonate production became negative varied across habitats (Figure H in S1 File), with the inner reefs of Palau showing the highest values (21%), with the outer and patch reefs of Palau, and the outer and inner reefs of Yap showing 10.2%, 11.8%, 9.5%, and 11.5%, respectively (Fig 5). These results suggest that some inner reefs tend to produce less carbonate than patch and outer reefs, which has also been observed on reefs in the Maldives [43]. These differences among reefs stem from differences in species composition, colony morphologies, and higher rates of erosion nearshore. For example, some inner reefs of Palau support extensive stands of arborescent colonies, for example *Anacropora* species. While *Anacropora* is a relatively rare coral genus throughout the Indo-Pacific, it has low density, and its branches are widely spaced, and therefore *Anacropora* does not produce high quantities of carbonate per unit area (for example at Site 11 in Palau, Figure A in S1 File). These results also suggest that because some inner reefs produce on average less carbonate than other reef types, they also need higher coral cover to produce the same amount of carbonate as patch and outer reefs.

The rates of carbonate production in the present study are considerably higher than contemporary rates of carbonate production estimated in the Caribbean. Perry *et al*. [6] estimated that on average Caribbean reefs had carbonate production rates at 3.7 kg CaCO_3_ m^−2^ yr^−1^, with many reefs displaying zero-net production, or even net erosion. These low rates are largely a consequence of a reduction of primary reef-builders in the Caribbean, including *Acropora palmata*, *Acropora cervicornis*, and *Orbicella* species, which have been diminished by frequent thermal stress and disease [44]. Indeed, losing the dominant reef builders of a system is problematic. Still, the western Pacific reefs appear to show some redundancy to species loss, which could confer resilience. Although the majority of carbonate production on both islands can be attributed to <10% of the local coral species, the coral species accreting the most carbonate differed somewhat between islands. Although *Porites lobata*, *Porites cylindrica*, and *Acropora formosa* (*muricata*) were dominant reef-builders on Palau and Yap, *Porites rus* was also dominant in Palau, particularly on inner reefs, and *Acropora palifera* was a dominant reef builder in Yap, particularly in the shallow lagoon habitat. The islands of Palau and Yap are geographically adjacent, yet history, geographic circumstance, and chance events may afford an advantage of one coral species over another, although essentially playing a similar role in reef-building across similar habitats. Therefore, the diverse western Pacific reefs, and the inter-changeability among some coral species, may provide some resilience to climate-change related disturbances compared with reefs in the Caribbean, which have lost many of their major reef-building corals.

The rate of overall reef carbonate production in the present study averaged 9.7 kg CaCO_3_ m^-2^ y^-1^ for both islands, which translates to a vertical growth rate of 7.9 mm each year (Eq 14). These rates of carbonate production agree with geological records from other reefs in the western and central Pacific [10,11]. At the most favorable localities in the present study, carbonate production rates were estimated at almost 20 kg CaCO_3_ m^-2^ yr^-1^, or 12.2 mm per year of vertical growth. The average rate of sea-level rise is expected to increase substantially from 2 mm to 9 mm a year, into the 21^st^ century [45–47], depending on the climate change scenario. Therefore, rates of contemporary carbonate reef production in Palau and Yap indicate that reefs will ‘keep up’ [7] with sea-level rise, under a representation concentration pathway (RCP) of 2.6 scenario, which predict rates of sea-level rise of 5 mm year [46]. Even under a RCP of 4.5 and 6, the reefs could keep up with expected rates of sea-level rise of 6.5 mm y^-1^ and 6.7 mm y^-1^. However, few reefs are expected to keep up with a RCP 8.5 scenario, of 9 mm y^-1^.

The capacity to keep up with sea-level rise will however depend of a number of conditions, including cyclone disturbance, sea-water temperature increases, land-use changes, sediment load, water quality, and ocean acidification. The cyclones that passed near Palau in 2012 and 2013 clearly reduced the reef-building capacity of the eastern slopes of Palau by approximately 2–3 kg CaCO_3_ m^-2^ y^-1^, even four years after impact. Reefs have the capacity to recover rapidly from disturbances [48], if disturbances are infrequent. However, cyclones have recently become more common in the tropics, where they were historically considered rare events [8]. Keeping up with projected sea-level rise may become problematic as storm patterns change from their historical trajectories [8], and cyclones become more intensive.

Thermal stress events are also becoming more frequent and intensive [46,49], reducing the capacity of reefs to accrete calcium carbonate, which in turn impairs the capacity of reefs to keep up with sea-level rise [6, 20]. Tanzil *et al*. [50] showed that for every 1°C increase in sea-water temperature, rates of coral growth would be reduced by 41–56%. Such reductions in rates of coral growth will reduce the rates at which reefs will be able to vertically accrete and keep up with sea-level rise. Similarly, van Woesik *et al*. [20] showed a decline in *Porites* growth rates above 29.5°C, which were incorporated into subsequent reef-accretion models. Their models showed that reefs in Palau might be able to keep up with sea-level rise under Representative Concentration Pathways (RCPs) 2.6, 4.5, and 6. However, under an extreme RCP of 8.5, the reef models showed that they were unlikely to keep up with sea-level rise. Yet, whether reefs will be able to keep up with modern sea-level rise will clearly depend on geographic differences in rates of change in ocean temperatures [51], and local-human disturbances, including land-use change and pollution [52–55].

Land-use change can cause loss of reef-building corals [53,54]. High turbidity, high nutrient concentrations, and high sediment loads are all associated with mismanaged lands, and all lead to coral loss [54]. Such conditions can also lead to increases in macro- and micro-borers, which further reduce reef-building capacity [14,16]. Poor land-use practices have long been known to increase the number of filter feeders on reefs, and can switch a reef from an autotrophic to a heterotrophic system under extreme conditions [55]. We noticed considerable coral loss at one of the nearshore reefs at Yap, north of Runn’uw. We learned that this reef had been harvested for coral materials to build a road four years previously. Here the rates of carbonate production were among the lowest in both islands, just above 1 kg CaCO_3_ m^-2^ yr^-1^, which gives warning to such practices and the long-term negative influences that coral harvesting has on carbonate production rates.

The uptake of carbon dioxide (CO_2_) by the oceans also potentially affects reef accretion rates by shifting the ocean’s acid-base balance toward a lower pH [56]. Oceanic pH has already decreased by 0.1 pH units since the 18th century [46], and is expected to drop by another 0.2–0.4 pH units by 2100. However, several studies have shown that corals are unaffected by external carbonate ion concentrations, because they have the capacity to up-regulate internal pH, through a hydrogen-pump mechanism, in their calicoblastic layer [57–59]. A recent study in Palau even showed high coral diversity, high coral cover, and relatively normal calcification rates (0.9 ± 0.02 g CaCO_3_ cm^-3^ yr^-1^) under chronically low pH and saturation state [60,61]. Indeed, McCulloch *et al*. [59] showed that by modifying their internal chemistry live corals might buffer themselves from ocean acidification. Therefore, ocean acidification may not be necessarily a live-coral problem [62]. Still, bare coral-colony skeletons, with no live tissue, have no internal buffering capacity and are susceptible to ocean acidification and subsequent erosion of carbonate substrate [63–65]. Therefore, chemical erosion of bare carbonate under reduced pH may become a significant player in carbonate budgets under severe climate-change scenarios.

## Conclusions

As in the past, rates of reef accretion in the future will depend on the persistence of reef-framework-building coral species, and on their capacity to accrete calcium carbonate faster than the various processes of erosion and dispersion. Importantly, reef structures protect tropical island nations from storm waves, and therefore protecting these bio-accreting systems is critical as sea levels continue to rise and the oceans continue to warm. Yet, whether reefs in Palau and Yap will be able to keep up with continued sea-level rise is largely dependent on future rates of sea-level rise, on future responses of reef accreting organisms to ocean warming, on controlling local pollution, and on the judicious management of land-use change. Our study suggested that because some inner reefs produce on average less carbonate than other reef types, these inner reefs therefore need higher coral cover to produce the same amount of carbonate as patch and outer reefs. In conclusion, the present study suggests that nearshore reefs are more vulnerable to sea-level rise than other reef habitats, which stresses the need for effective land-use practices as the climate continues to change.

## Supporting information

S1 Data The file is a compressed file that contains all the raw data files as Excel spreadsheet tables and all the R scripts that produced the figures in the manuscript; note that there is a Read me file, which explains the content of each file. (RAR)

S1 File The file contains supporting tables and figures not in the main manuscript. The file includes a table on morphological adjustments for corals that were used in the calculations, laboratory measured skeletal densities used in the calculations, the contribution of each coral and herbivorous fish species per island, kriged maps of the contribution of several fishes and echinoids to carbonate erosion, and the recorded coral cover partitioned by country, habitat, and site.
(DOCX)
