# Supplementary material for: Keeping up with sea-level rise: Carbonate production rates in Palau and Yap, western Pacific Ocean
Source: PLoS One. 2018 May 8;13(5):e0197077. doi: 10.1371/journal.pone.0197077 (PMC5940225; doi:10.1371/journal.pone.0197077)
Supplement: S1 File — The file includes a table on morphological adjustments for corals that were used in the calculations, laboratory measured skeletal densities used in the calculations, the contribution of each coral and herbivorous fish species per island, kriged maps of the contribution of several fishes and echinoids to carbonate erosion, and the recorded coral cover partitioned by country, habitat, and site. (DOCX) [file pone.0197077.s002.docx]

Keeping up with sea-level rise: carbonate production rates in Palau and Yap, western Pacific Ocean

R. van Woesik and C. Cacciapaglia


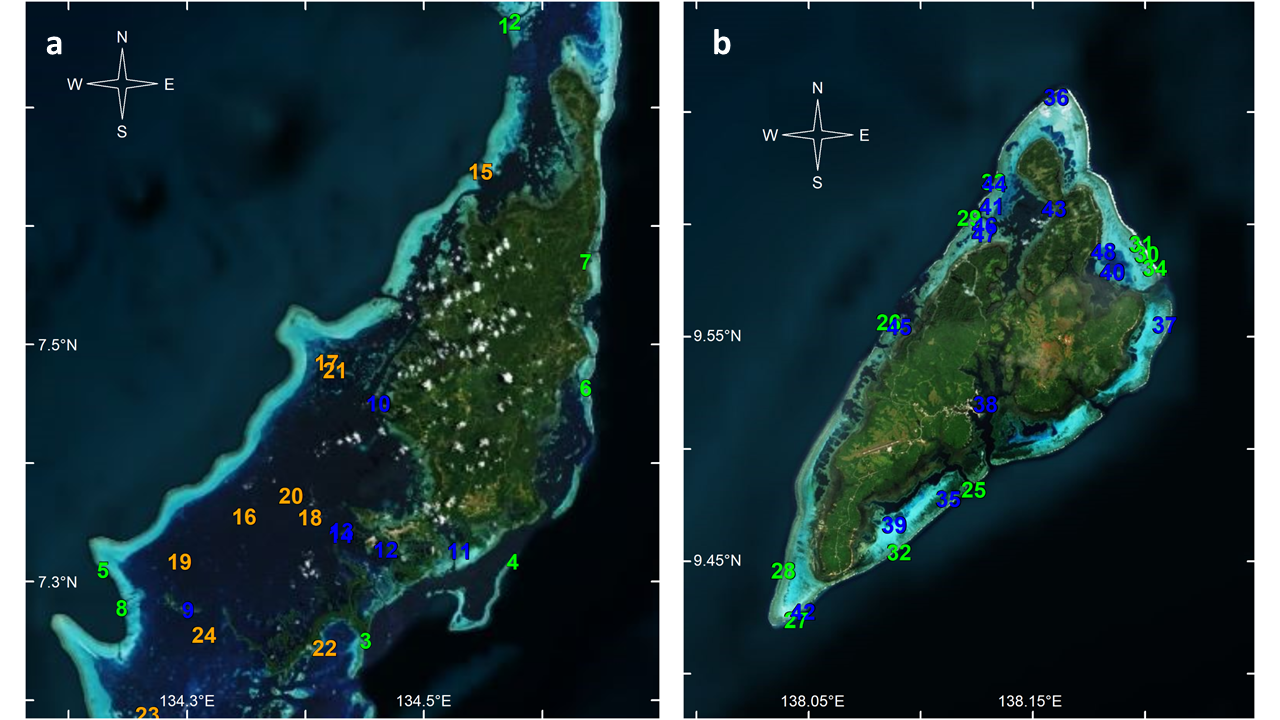


**Figure A.** Study sites in (a) Palau and (b) Yap with numbers corresponding to Figure 2. Green numbers indicate outer reefs, blue numbers indicate inner reefs, and orange numbers indicate patch reefs.

*Coral morphologies*

Since we were interested in the planar cover of corals, and many corals are not flat or massive, we calculated the morphological adjustment coefficient, *m*, for different coral growth forms, including massive, encrusting, corymbose, caespitose, and branching morphologies (Table A). We also used the Palau coral collection, and measured the densities of 64 corals from the coral collection (Table B). Densities (g cm^-3^) of coral colonies were measured using the Archimedes principle. The densities were validated using Pratchett et al. (2015) dataset. These allometric relationships allowed us to determine the adjustment coefficients that relate the morphologies of coral colonies with the potential rate of carbonate deposition. We used these morphological adjustments and the chord size along the line transects, along with skeletal density (d_i_) (expressed as g cm^-3^) of each species *i,* and *g*_i_ the linear growth rates (from the literature, particularly from the extensive review by Pratchett et al. 2015) to estimate carbonate production (kg CaCO_3_ m^-2^ y^-1^).

**Table A.** Morphological adjustment coefficient, *m*, for the most common coral species in Palau and Yap.

| **Species** | **Length (m)** | **Height (m)** | **non-rugose area (length*height; m^2)** | **Volume (m^3)** | **Cross sectional area estimate (m^2)** | **Morphological adjustment ratio** | **Morphology** | **Source** |
| --- | --- | --- | --- | --- | --- | --- | --- | --- |
| *Acropora palifera* | 0.8 | 0.39 | 0.312 | 0.028 | 0.111479793 | 0.357307029 | submassive | field |
| *Porites cylindrica* | 1.03 | 0.435 | 0.44805 | 0.0464 | 0.15611194 | 0.348425265 | digitate | field |
| *Acropora nobilis* | 0.812 | 0.16 | 0.12992 | 0.003 | 0.025148088 | 0.193565947 | branching | field |
| *Acropora pulchra* | 0.14 | 0.08 | 0.0112 | 0.000021 | 0.000920245 | 0.082164769 | branching | lab |
| *Acropora divaricata* | 0.064 | 0.0815 | 0.005216 | 0.000102 | 0.002639313 | 0.50600328 | corymbose | lab |
| *Acropora nasuta* | 0.0713 | 0.0734 | 0.00523342 | 0.000071 | 0.002072986 | 0.396105412 | corymbose | lab |

Finding cross sectional area (m^2) to length of corals, for example, *Acropora palifera*, L=0.8, h=0.39, V= 0.028, *Porites cylindrica*, L=1.03, h=0.435, V= .0464064, using the following function in R: XAratio<-function(L,h,V){ r<-(V/((4/3)*pi))^(1/3) #take volume and assume shape is a sphere to find radius

xa<-pi*r^2 #find cross sectional area of assumed sphere given radius

A<-L*h #find the area of the coral(as if assuming the coral grows vertical like a massive)

return(xa/A) #return ratio between the rugose coral and the measured corals (rK)}

XAratio(L,h,V)

**Table B.** Estimates of skeletal density (d_i_) of 64 common corals in Palau (expressed as g ml, which is equal to g cm^-3^).

| **Family** | **Genus** | **Species** | **Weight** (g**)** | **Displacement (ml)** | **Density (g/ml)** | **Morphology** |
| --- | --- | --- | --- | --- | --- | --- |
| Acroporidae | *Acropora* | *awi* | 41.90 | 37 | 1.132 | caespitose |
| Acroporidae | *Montipora* | *angulata* | 55.25 | 44 | 1.256 | branching |
| Acroporidae | *Acropora* | *cerialis* | 160.22 | 91 | 1.761 | corymbose |
| Acroporidae | *Acropora* | *nobilis* | 40.88 | 23 | 1.777 | branching |
| Acroporidae | *Acropora* | *digitifera* | 21.54 | 12 | 1.795 | digitate |
| Acroporidae | *Acropora* | *valenciennesi* | 45.69 | 25 | 1.828 | corymbose |
| Acroporidae | *Montipora* | *montasteriata* | 24.75 | 13 | 1.904 | encrusting |
| Acroporidae | *Acropora* | *hyacinthus* | 116.55 | 60 | 1.943 | tabulate |
| Acroporidae | *Acropora* | *echinata* | 37.28 | 16 | 2.330 | caespitose |
| Acroporidae | *Acropora* | *subglabra* | 82.38 | 33 | 2.496 | caespitose |
| Acroporidae | *Acropora* | *nasuta* | 26.52 | 10 | 2.652 | corymbose |
| Acroporidae | *Acropora* | *samoensis* | 60.47 | 20 | 3.024 | corymbose |
| Acroporidae | *Acropora* | *formosa* | 56.12 | 15 | 3.741 | branching |
| Agaricidae | *Pavona* | *venosa* | 148.05 | 105 | 1.410 | encrusting |
| Agaricidae | *Pachyseris* | *speciosa* | 29.70 | 18 | 1.650 | foliose |
| Agaricidae | *Leptoseris* | *explanata* | 52.30 | 29 | 1.803 | foliose |
| Astrocoeniidae | *Stylocoeniella* | *armata* | 72.01 | 40 | 1.800 | encrusting |
| Astrocoeniidae | *Palauastrea* | *ramosa* | 92.71 | 36 | 2.575 | branching |
| Dendrophyllidae | *Turbinaria* | *frondens* | 90.54 | 44 | 2.058 | foliose |
| Dendrophyllidae | *Turbinaria* | *mesenterina* | 138.27 | 72 | 1.920 | foliose |
| Merulinidae | *Favia* | *helianthoides* | 52.75 | 12 | 4.396 | massive |
| Merulinidae | *Favia* | *matthaii* | 110.48 | 87 | 1.270 | massive |
| Merulinidae | *Favites* | *abdita* | 271.52 | 177 | 1.534 | massive |
| Merulinidae | *Cyphastrea* | *chalcidicum* | 281.00 | 169 | 1.663 | encrusting |
| Merulinidae | *Platygyra* | *pini* | 202.99 | 117 | 1.735 | massive |
| Merulinidae | *Favia* | *stelligera* | 266.67 | 149 | 1.790 | massive |
| Merulinidae | *Montastrea* | *curta* | 59.65 | 29 | 2.057 | massive |
| Merulinidae | *Leptoria* | *phrygia* | 119.96 | 58 | 2.068 | massive |
| Merulinidae | *Favites* | *halicora* | 169.97 | 80 | 2.125 | massive |
| Merulinidae | *Caulastrea* | *furcata* | 93.49 | 41 | 2.280 | submassive |
| Merulinidae | *Leptastrea* | *pruinosa* | 76.85 | 30 | 2.562 | encrusting |
| Merulinidae | *Favites* | *pentagona* | 159.00 | 61 | 2.607 | massive |
| Merulinidae | *Goniastrea* | *palauensis* | 32.54 | 6 | 5.423 | massive |
| Fungiidae | *Fungia* | *paumotensis* | 170.24 | 89 | 1.913 | solitary |
| Fungiidae | *Herpolitha* | *limax* | 118.81 | 59 | 2.014 | solitary |
| Fungiidae | *Fungia* | *costulata* | 34.54 | 16 | 2.159 | solitary |
| Fungiidae | *Sandolithia* | *robusta* | 71.82 | 33 | 2.176 | solitary |
| Fungiidae | *Cycloseris* | *vaughani* | 43.51 | 13 | 3.347 | solitary |
| Helioporidae | *Heliopora* | *coerulea* | 5.75 | 3 | 1.917 | submassive |
| Merulinidae | *Hydnophora* | *bonsai* | 43.73 | 28 | 1.562 | submassive |
| Merulinidae | *Merulina* | *ampliata* | 28.79 | 15 | 1.919 | encrusting |
| Merulinidae | *Hydnophora* | *exesa* | 38.85 | 18 | 2.158 | submassive |
| Merulinidae | *Merulina* | *scabricula* | 19.60 | 9 | 2.178 | foliose |
| Milleporidae | *Millepora* | *tenella* | 49.74 | 29 | 1.715 | branching |
| Mussidae | *Lobophyllia* | *hemprichi* | 212.92 | 190 | 1.121 | massive |
| Mussidae | *Hydrophora* | *microconus* | 234.95 | 162 | 1.450 | massive |
| Oculinidae | *Galaxea* | *astreata* | 25.01 | 13 | 1.924 | massive |
| Oculinidae | *Galaxea* | *archelia* | 28.78 | 7 | 4.111 | branching |
| Pectiniidae | *Echinophyllia* | *costata* | 29.75 | 18 | 1.653 | encrusting |
| Pectiniidae | *Oxypora* | *lacera* | 24.84 | 8 | 3.105 | foliose |
| Pocilloporidae | *Pocillopora* | *damicornis* | 136.67 | 71 | 1.925 | corymbose |
| Pocilloporidae | *Stylophora* | *pistillata* | 119.97 | 55 | 2.181 | corymbose |
| Pocilloporidae | *Seriatopora* | *hystrix* | 51.61 | 20 | 2.581 | branching |
| Pocilloporidae | *Pocillopora* | *verrucosa* | 216.90 | 80 | 2.711 | corymbose |
| Poritidae | *Alveopora* | *allingi* | 115.52 | 108 | 1.070 | encrusting |
| Poritidae | *Goniopora* | *columna* | 176.03 | 135 | 1.304 | massive |
| Poritidae | *Goniopora* | *planulata* | 106.40 | 78 | 1.364 | massive |
| Poritidae | *Porites* | *annae* | 97.68 | 70 | 1.395 | submassive |
| Poritidae | *Porites* | *rus* | 84.30 | 60 | 1.405 | submassive |
| Poritidae | *Porites* | *cylindrica* | 28.21 | 18 | 1.567 | branching |
| Poritidae | *Porites* | *lobata* | 483.91 | 287 | 1.686 | massive |
| Siderastreidae | *Psammocera* | *superficialis* | 12.04 | 7 | 1.720 | encrusting |
| Siderastreidae | *Psammocera* | *vaughani* | 287.00 | 153 | 1.876 | encrusting |
| Trachyphyllidae | *Trachyphillia* | *geoffroyi* | 133.76 | 61 | 2.193 | solitary |

**
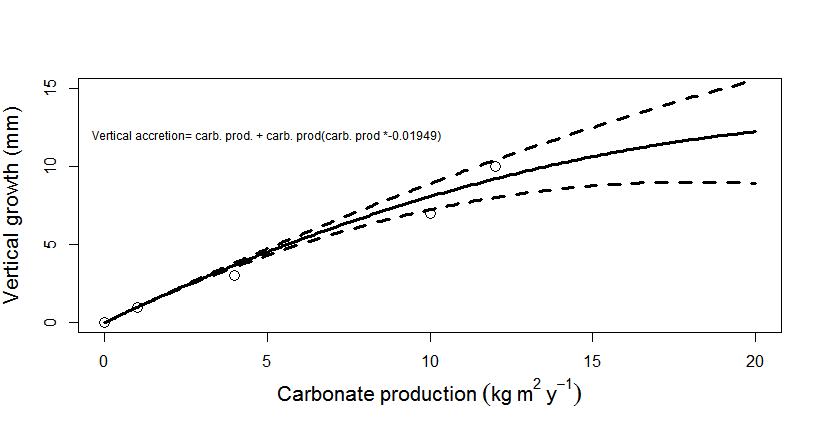
**

**Figure B.** Estimated vertical accretion potential of a reef as a function of carbonate production (kg m^2^ y^-1^), with the best fit function using data from references [11, 26]. The dark line is the best fit line for equation 14, and the dashed lines are the 95% confidence intervals.

**Figure C.** Contribution of carbonate production (kg CaCO_3_ m^-2^ y^-1^) by coral species in Palau and Yap.


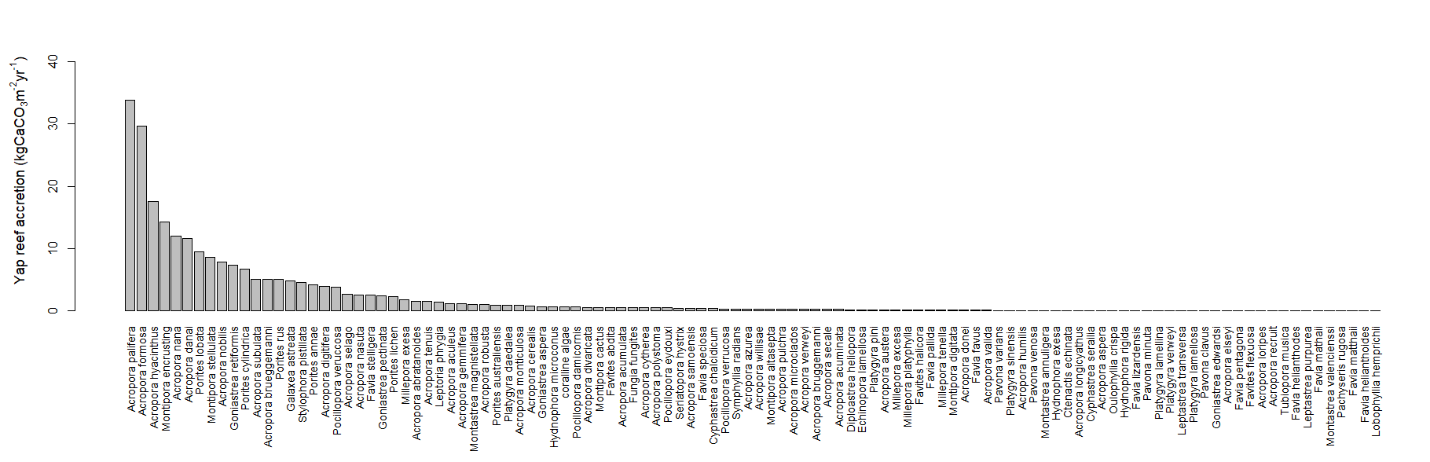

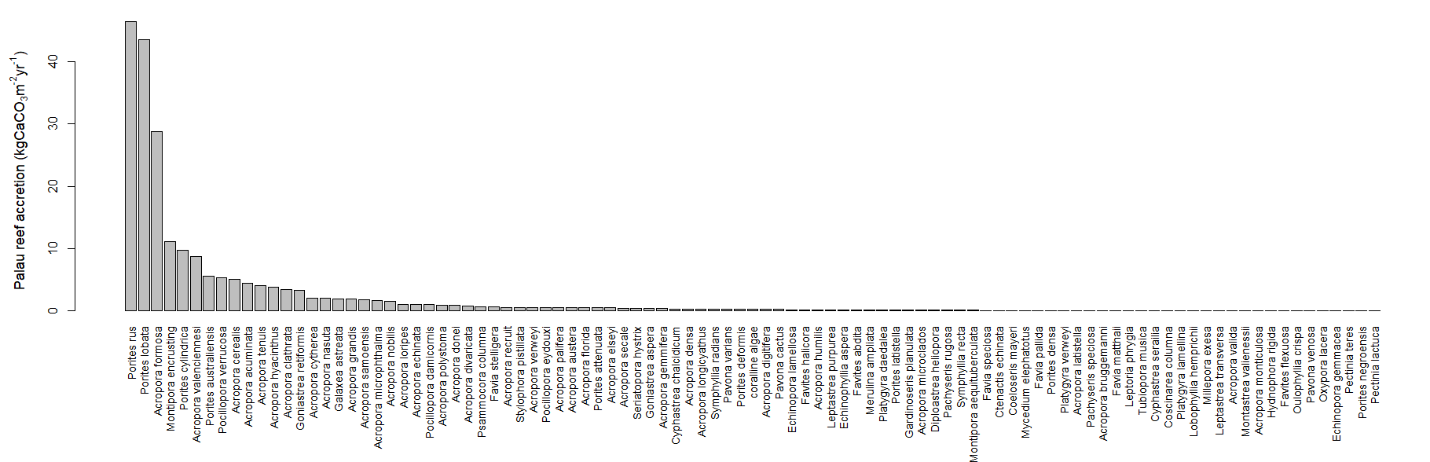


Palau reef accretion (kg CaCO_3_ m^-2^ yr^-1^)

Yap reef accretion (kg CaCO_3_ m^-2^ yr^-1^)

**a**

**b**

**
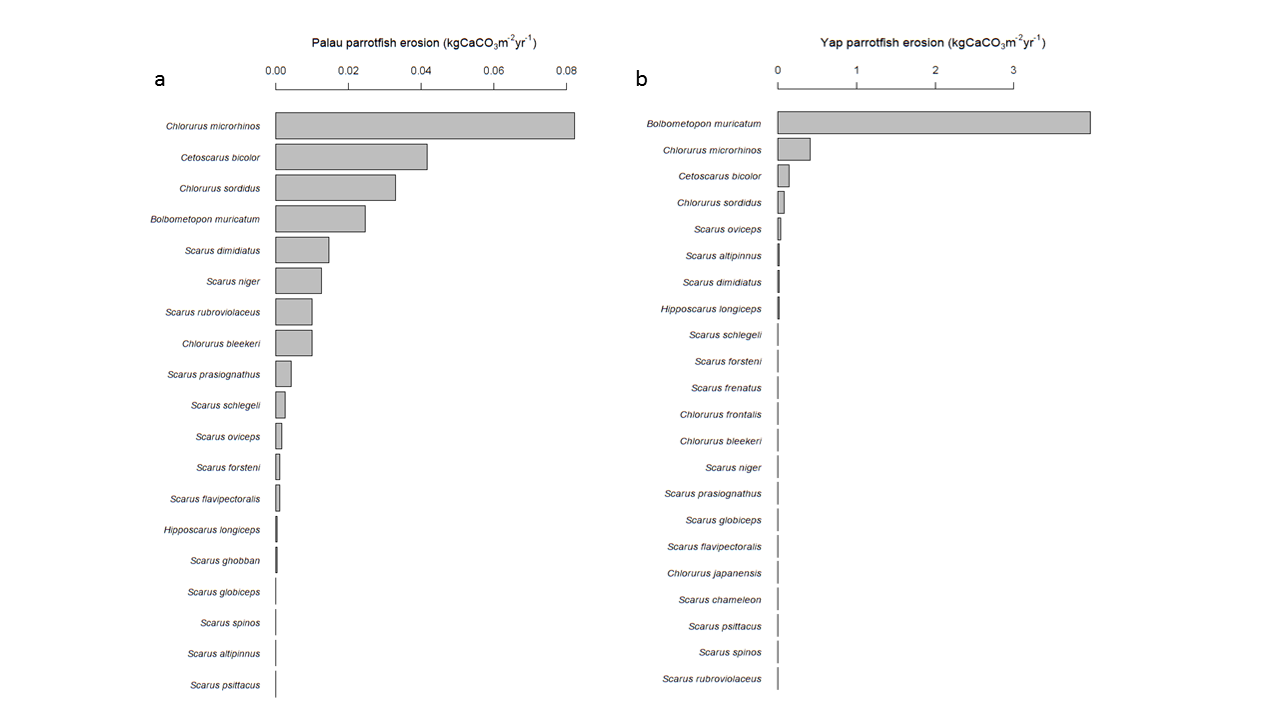
**

**Figure D.** Relative contribution of herbivorous fish bioerosion rates (kg CaCO_3_ m^-2^ yr^-1^) for Palau (a) and Yap (b).


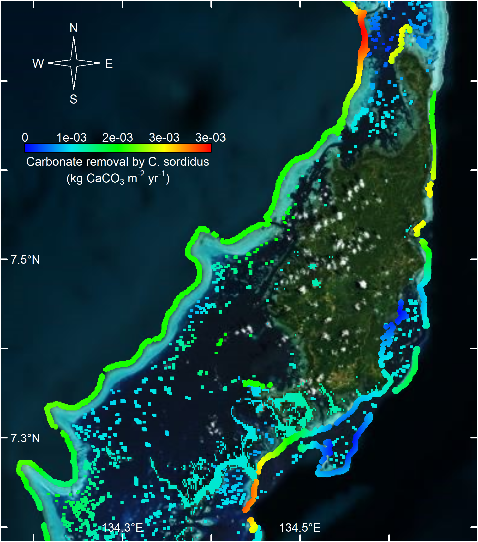

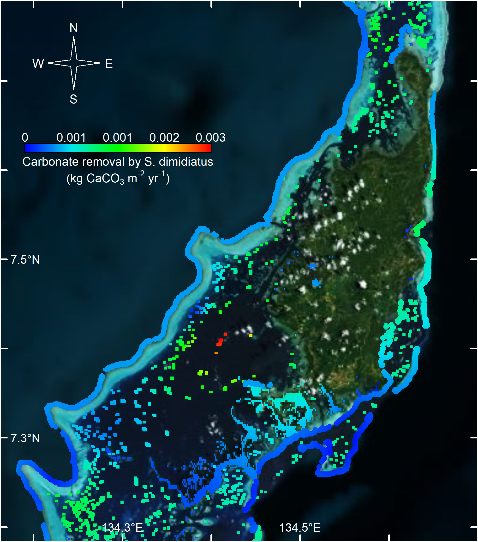

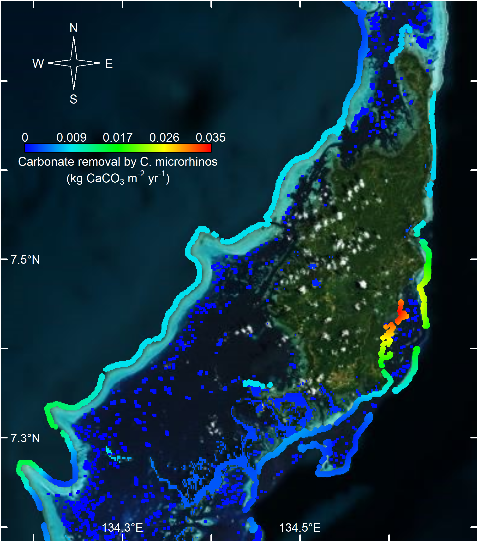

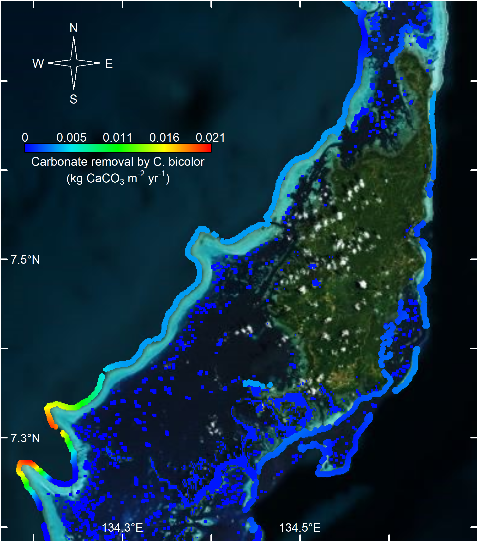

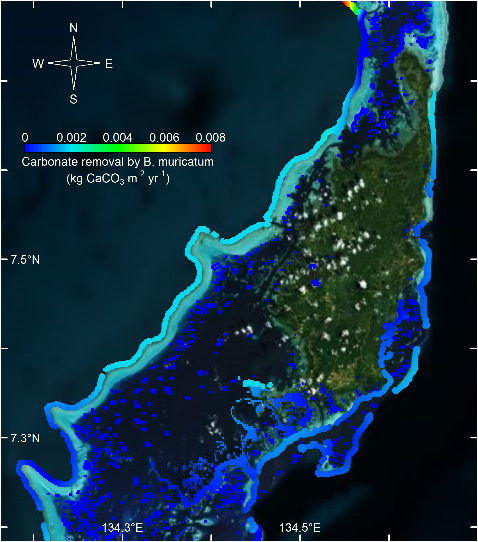


**a**

**b**

**c**

**e**

**d**

**Figure E**. Removal of carbonate (kg CaCO_3_ m^-2^ yr^-1^) by five species of parrotfish, kriged for Palau. Red indicates high rates of carbonate removal by parrotfish, and blue indicates low rates of carbonate removal by parrotfish, where (a) S. dimidiatus represents *Scarus dimidiatus*, (b) C. sordidus represents *Chlorurus sordidus*, (c) C. microrhinous represents *Chlorurus microrhinos*, (d) B. muricatum represents *Bolbometopon muricatum*, and (e) C. bicolor represents *Cetoscarus bicolor*.


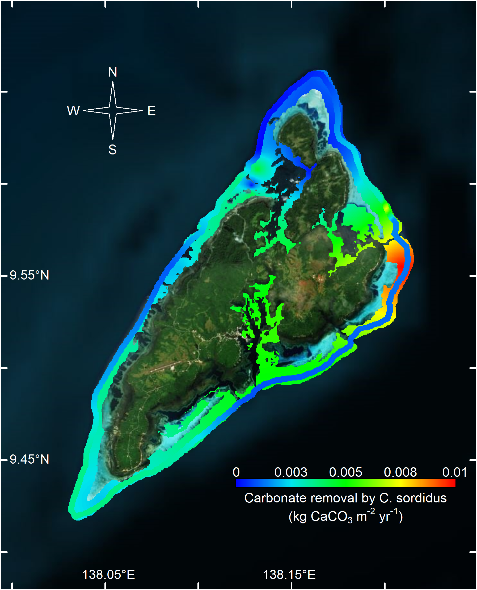

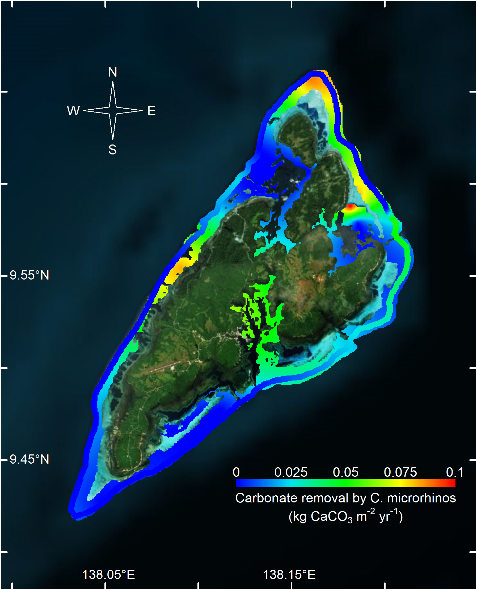

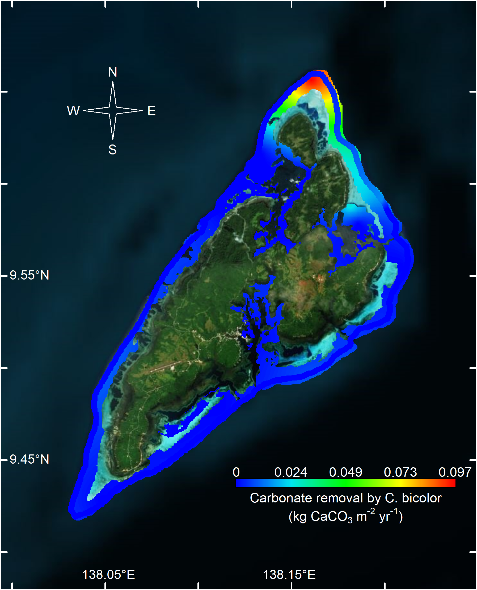

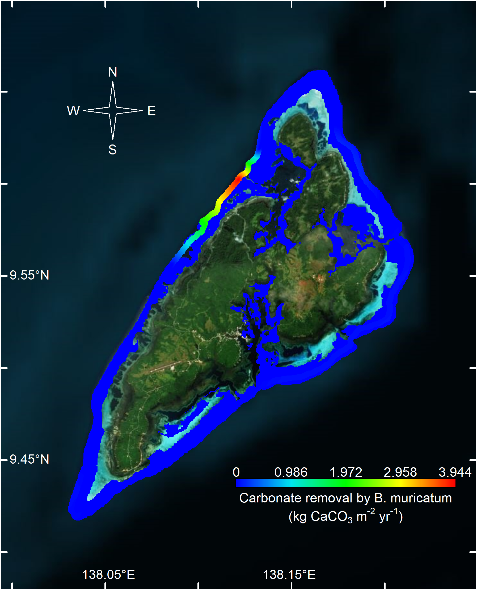

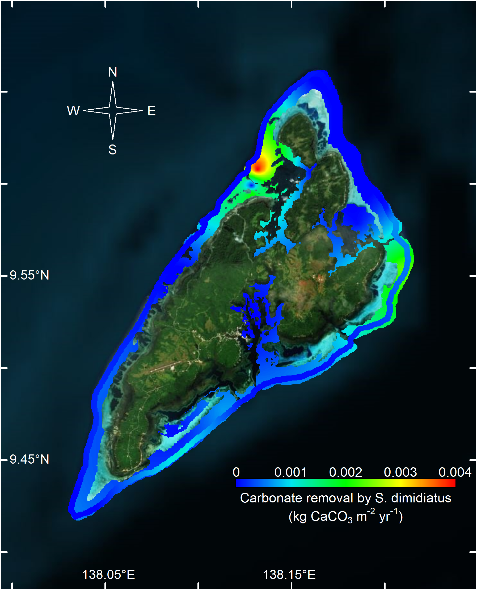


**a**

**b**

**c**

**e**

**d**

**Figure F**. Removal of carbonate (kg CaCO_3_ m^-2^ yr^-1^) by parrotfish, kriged for Yap. Red indicates high rates of carbonate removal by parrotfish, and blue indicates low rates of carbonate removal by parrotfish, where (a) C. sordidus represents *Chlorurus sordidus*, (b) C. microrhinous represents *Chlorurus microrhinos*, (c) C. bicolor represents *Cetoscarus bicolor,* (d) B. muricatum represents *Bolbometopon muricatum ,* and (e) S. dimidiatus represents *Scarus dimidiatus*.

**
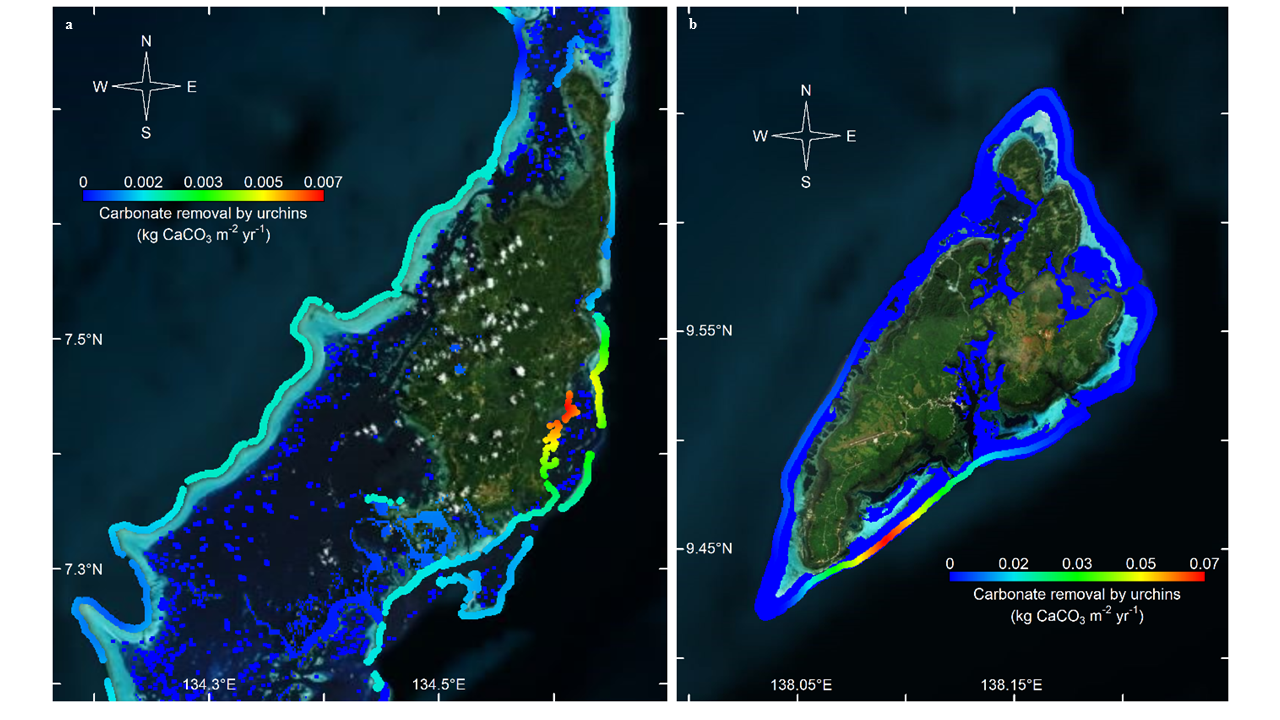
**

**Figure G.** Removal of carbonate (kg CaCO_3_ m^-2^ yr^-1^) by echinoids, kriged for Palau (a) and Yap (b). Red indicates high rates of carbonate removal by urchins, and blue indicates low rates of carbonate removal by urchins.

**Table C.** Echinoid erosion in kg CaCO_3_ m^-2^ yr^-1^ by island (kg CaCO_3_ m^-2^ y^-1^).

|  | *Echinometra* | *Diadema* | General |
| --- | --- | --- | --- |
| Palau | 0.0087999631 | 0.0001702375 | 0.0085764699 |
| Yap | 0.07030931 | 0.00000000 | 0.02916612 |

**
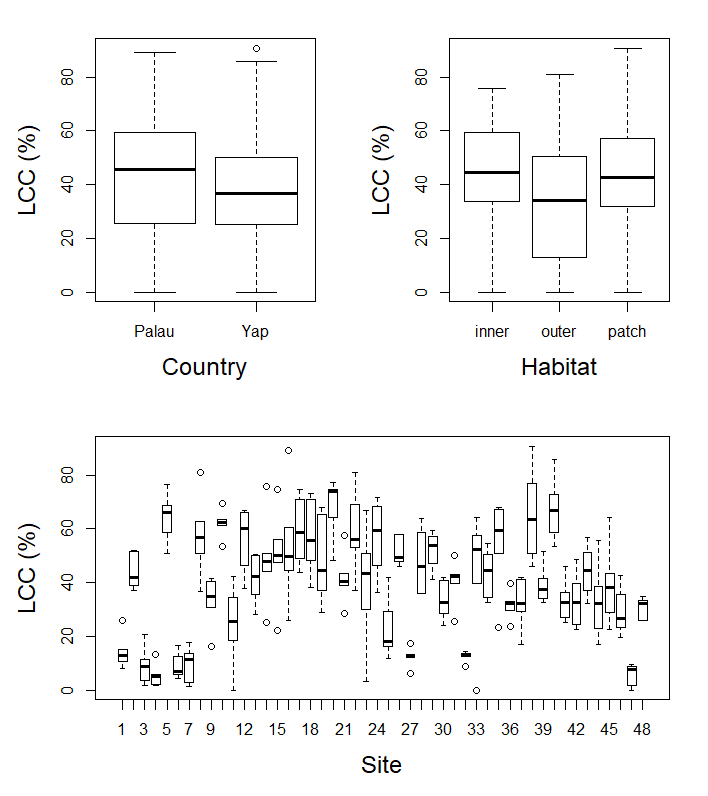
**

**Figure H.** Live coral cover (LLC) conditional on country (Palau and Yap), habitat, and site, where sites correspond to sites in Figure S1, where the thick horizontal lines are the medians, the box surrounding the medians are the first and third quartiles, the whiskers identify the range of the data, and the circles identify outliers.
